# Microbiota: A key factor affecting and regulating the efficacy of immunotherapy

**DOI:** 10.1002/ctm2.1508

**Published:** 2023-12-11

**Authors:** Yao Jiang, Dingjiacheng Jia, Yong Sun, Ning Ding, Liangjing Wang

**Affiliations:** ^1^ Department of Gastroenterology Second Affiliated Hospital of Zhejiang University School of Medicine Hangzhou China; ^2^ Institution of Gastroenterology Zhejiang University Hangzhou China; ^3^ Cancer Center Zhejiang University Hangzhou China

**Keywords:** cancer immunotherapy, gut microbiota, immune checkpoint inhibitors

## Abstract

**Background:**

Immunotherapy has made significant progress in cancer treatment; however, the responsiveness to immunotherapy varies widely among patients. Growing evidence has demonstrated the role of the gut microbiota in the efficacy of immunotherapy.

**Main body:**

Herein, we summarise the changes in the microbiota in different cancers under various immunotherapies. The microbial‐host signal transmission on immunotherapeutic responses and mechanisms associated with microbial translocation to tumours in the context of immunotherapy are also discussed. In addition, we have highlighted the clinical application value of methods for regulating the microbiota. Finally, we elaborate on the relationship between the microbiota, host and immunotherapy, and provide potential directions for future research.

**Conclusion:**

Different microbiota cause changes in the tumour microenvironment through microbial signals thereby affecting immunotherapy efficacy. Translocation of gut microbiota and the role of extraintestinal microbiota in immunotherapy deserve attention. Microbiota regulation is a novel strategy for combination therapy with immunotherapy. Although there are several aspects that deserve further refinement and exploration with regard to administration and clinical translation. Nevertheless, it is foreseeable that the microbiota will become an integral part of cancer treatment.

## Graphical abstract

1. The changes of microbiota in different cancers under various immunotherapies are summarised.
2. The mechanisms of bacterial translocation and by which microbiota influence efficacy of immunotherapy are discussed.
3. The strategies of regulating immunotherapy by gut microbiota modification are elaborated.
4. The interactions between immunotherapy, host and gut microbiota are illustrated.

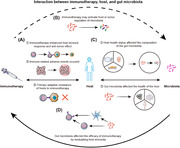

## 1 INTRODUCTION

As a significant breakthrough in cancer treatment, tumour immunotherapy could restart the normal immune responses and reduce immune escape and tolerance.^1^ A variety of immune checkpoint inhibitors (ICIs), such as cytotoxic T‐lymphocyte‐associated antigen 4 (CTLA‐4), programmed death protein‐1 (PD‐1) and its ligand, PD‐L1 antibody, have been broadly applied in clinical practice.^2^ However, limited response rates, lack of clinical response prediction markers and potential toxic side effects have hindered their application.^3^ Therefore, it is crucial to develop safe and effective methods to enhance immunotherapy.^4^ Bacteria, archaea, bacteriophages, eukaryotic viruses and fungi form the microbiota, which is widely distributed throughout the human body.^5^, ^6^ Microbiota‐host imbalance has been reported to be associated with various diseases, such as obesity, malnutrition, inflammatory bowel disease, neurological disorders and cancers.^7^, ^10^ Growing evidence has shown that the microbiota not only directly influences cancer development,^11^ but also plays an important role in immunotherapy.

Herein, we review the literatures on the influence of microbiota on the efficacy of immunotherapy, the microbial‐host signal transmission on immunotherapeutic responses and mechanisms associated with microbial translocation to tumours in the context of immunotherapy. In addition, we illustrate the interplay between the gut microbiota, immunotherapy and the host. Finally, we propose possibilities and future directions for improving the efficacy of ICIs by altering the microbiota.

## 2 THE MICROBIOTA PLAYS A ROLE IN CANCER IMMUNOTHERAPY

**FIGURE 1:**
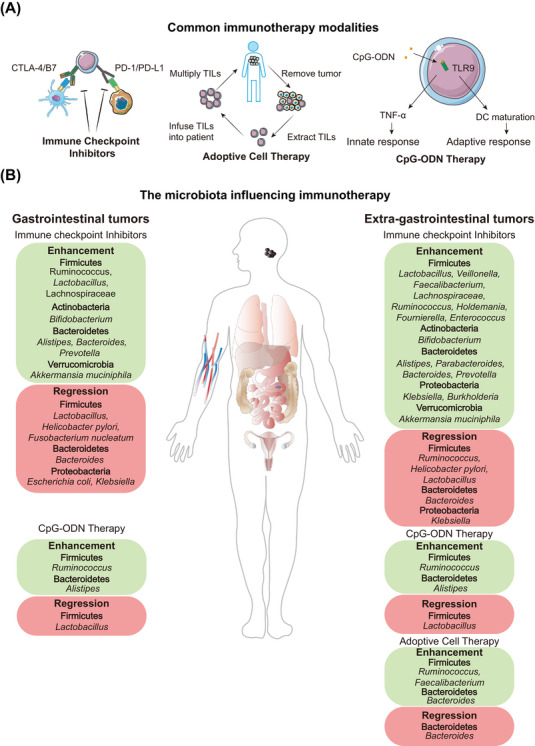
The microbiota plays a role in multiple cancer immunotherapy. (A) Effect of microbes on gastrointestinal tumours under different immunotherapy treatments. (B) Effect of microbes on extragastrointestinal tumours under different immunotherapy treatments.

Over the past few years, microbes have been implicated in immunotherapy for numerous malignancies in both animal models and human studies (Table 1, Figure 1).^12^, ^13^, ^14^, ^15^, ^16^, ^17^, ^18^, ^19^, ^20^, ^21^, ^22^, ^23^, ^24^, ^25^, ^26^, ^27^, ^28^, ^29^, ^30^, ^31^, ^32^, ^33^

**TABLE 1:** Change of microbiota in the immunotherapy of multiple cancers.

	Tumour type	Immuno‐therapy	Influence on therapy	Phylum	Family	Genus	Bacterium	Host	Particular year	Ref
Extra‐gastrointestinal tumours	Melanoma	Anti‐ PD‐1/PD‐L1 Therapy	Enhancement	Actinobacteria	Bifidobacteriaceae	Bifidobacterium	*Bifidobacterium abreve*	Mice	2015	[13]
							*Bifidobacterium adolescentis*			
							*Bifidobacterium longum*			
							*Bifidobacterium adolescentis*	Reactive patients	2018	[20]
				Bacteroidetes	Tannerellaceae	Parabacteroides	*Parabacteroides merdae*			
				Firmicutes	Lactobacillaceae	Ligilactobacillus	*Lactobacillus*			
					Veillonellaceae	Veillonella	*Veillonella parvula*			
					Enterococcaceae	Enterococcus				
					Oscillospiraceae	Faecalibacterium	*Faecalibacterium*		2018	[21]
					Lachnospiraceae	Dorea	*Dorea formicigenerans*		2017	[19]
				Proteobacteria	Enterobacteriaceae	Klebsiella	*Klebsiella pneumoniae*		2018	[20]
			Regression	Firmicutes	Oscillospiraceae	Ruminococcus	*Ruminococcus*	Non‐reactive patients	2018	[20]
				Bacteroidetes	Bacteroidaceae	Bacteroides	*–*	Reactive patients	2018	[21]
		Anti‐CTLA‐4 Therapy	Enhancement	Bacteroidetes	Bacteroidaceae	Bacteroides	*Bacteroides fragilis*	Mice	2015	[14]
				Proteobacteria	Burkholderiaceae	Burkholderia	*Burkholderia cepacia*			
				Firmicutes	Lachnospiraceae	Lachnospiraceae	*–*	Reactive Patients	2017	[22]
					Oscillospiraceae	Ruminococcus	*Ruminococcus*			
						Faecalibacterium	*Faecalibacterium*			
									2020	[23]
			Regression	Bacteroides	Bacteroidaceae	Bacteroides	*–*	Reactive patients	2017	[22]
		Combination anti‐PD1 and anti‐CTLA4 (CICB))	Enhancement	Firmicutes	Oscillospiraceae	Faecalibacterium	*Faecalibacterium prausnitzii*	Reactive patients	2017	[19]
					Erysipelotrichaceae	Holdemania	*Holdemania filiformis*			
					Oscillospiraceae	Fournierella	*Fournierella massiliensis*		2021	[24]
				Bacteroidetes	Bacteroidaceae	Bacteroides	*Bacteroides thetaiotamicron*		2017	[19]
							*Bacteroides stercoris*		2021	[24]
					Tannerellaceae	Parabacteroides	*Parabacteroides distasonis*			
			Regression	Firmicutes	Lactobacillaceae	Ligilactobacillus	*Lactobacillus rogosae*	Non‐reactive patients	2021	[24]
				Proteobacteria	Enterobacteriaceae	Klebsiella	*Klebsiella aerogenes*			
		Anti‐ PD‐1 OR Anti‐CTLA‐4	Regression	Firmicutes	*Helicobacter pylori*			Mice	2021	[25]
		CpG‐ODN Therapy	Enhancement	Firmicutes	Gram‐negative LPS‐producing bacteria			Mice	2013	[15]
					Oscillospiraceae	Ruminococcus	*Ruminococcus*			
				Bacteroidetes	Rikenellaceae	Alistipes	*Alistipes shahii*			
			Regression	Firmicutes	Lactobacillaceae	Ligilactobacillus	*Lactobacillus*			
	NSCLC	Anti‐ PD‐1/PD‐L1 Therapy	Enhancement	Bacteroidetes	Bacteroidaceae	Bacteroides	*–*		2022	[26]
				Verrucomicrobia	Akkermansiaceae	Akkermansia	*–*			
				Firmicutes	Lachnospiraceae	Blautia	*–*			
				Actinobacteria	Bifidobacteriaceae	Bifidobacterium	*Bifidobacterium longum*	Reactive patients	2019	[28]
				Bacteroidetes	Rikenellaceae	Alistipes	*Alistipes putredinis*			
					Prevotellaceae	Prevotella	*Prevotella corpri*			
				Verrucomicrobia	Akkermansiaceae	Veillonella	*Akkermansia muciniphila*		2018	[27]
									2022	[26]
				Firmicutes	Oscillospiraceae	Ruminococcus	*–*		2022	[26]
						Faecalibacterium	*–*			
			Regression	Firmicutes	Oscillospiraceae	Ruminococcus	*Ruminococcus obeum*	Reactive patients	2019	[28]
			Regression	–	–	–	*Helicobacter pylori*	Patients	2021	[25]
	RCC	Anti‐ PD‐1/PD‐L1 Therapy	Enhancement	Ibid			*Ibid*	Reactive patients	2019	[28]
				Verrucomicrobia	Akkermansiaceae	Veillonella	*Akkermansia muciniphila*		2018	[27]
			Regression	Ibid			*Ibid*	Non‐Reactive patients	2019	[28]
		Anti‐ PD‐1 OR CICB	Enhancement	Verrucomicrobia	Akkermansiaceae	Veillonella	*Akkermansia muciniphila*	Reactive patients	2020	[29]
				Bacteroidetes	Prevotellaceae	Prevotella	*Prevotella copri*			
	Hematologic Tumours	allo‐HCT	Enhancement	Firmicutes	Eubacteriaceae	Eubacterium	*Eubacterium limosum*	patients	2017	[18]
					Lachnospiraceae	Blautia	*Blautia*	Patient	2015	[17]
		anti‐CD19 CAR T cell therapy	Enhancement	Firmicutes	Oscillospiraceae	Ruminococcaceae	*Ruminococcus bromii*	Reactive patients	2022	[30]
						Faecalibacterium	*aecalibacterium prausnitzii*			
	cervical cancer	ACT	Enhancement	Bacteroidetes	Bacteroidaceae	Bacteroides	–	Mice	2018	[31]
					Tannerellaceae	Parabacteroides	–			
				Gram‐negative LPS‐producing bacteria					2007	[12]
			Regression	Bacteroidetes	Bacteroidales	Muribaculaceae S24‐7	–	Mice	2018	[31]
	sarcoma	Anti‐CTLA‐4 Therapy	Enhancement	Ibid			Ibid	Mice	2015	[14]
	lymphoma	CpG‐ODN Therapy	Enhancement	Ibid			Ibid	Mice	2013	[15]
			Regression	Ibid			Ibid			
Intra‐gastrointestinal tumours	CRC	Anti‐CTLA‐4 Therapy	Enhancement	Ibid			Ibid	Mice	2015	[14]
	CRC	CpG‐ODN Therapy		Ibid			Ibid	Mice	2013	[15]
	CRC		Regression	Ibid			Ibid	Mice	2013	[15]
	CRC	Anti‐PD‐1/PD‐L1 Therapy	Enhancement	–	–	–	Fusobacterium nucleatum	Patients	2021	[33]
	CRC	Anti‐ PD‐1 OR Anti‐CTLA‐4	Regression	Ibid			Ibid	Mice	2021	[25]
	HCC	Anti‐PD‐1/PD‐L1 Therapy	Enhancement	Actinobacteria	Bifidobacteriaceae	Bifidobacterium	Bifidobacterium dentium	Reactive patients	2019	[33]
				Firmicutes	Lactobacillaceae	Ligilactobacillus	Lactobacillus			
					Oscillospiraceae	Ruminococcus	Ruminococcus obeum			
				Verrucomicrobia	Akkermansiaceae	Veillonella	Akkermansia muciniphila			
			Regression	Proteobacteria	Enterobacteriaceae	Escherichia	Escherichia coli	Nonn‐reactive patient		
					Enterobacteriaceae	Klebsiella	Klebsiella pneumoniae			
	Gastrointestinal Cancer	Anti‐ PD‐1/PD‐L1 Therapy	Enhancement	Bacteroidetes	Prevotellaceae	Prevotella	Prevotella	Reactive patients	2020	[16]
				Firmicutes	Oscillospiraceae	Ruminococcus	–			
					Lachnospiraceae	–	–			
				Verrucomicrobia	Akkermansiaceae	Veillonella	Akkermansia muciniphila			
			Regression	Bacteroidetes	Bacteroidaceae	–	–	ReactivePatients	2020	[16]

### 2.1 Higher taxonomic levels may be more suitable as microbial predictors of immunotherapy

When a strain affects the efficacy of immunotherapy, other members of the genus or higher taxonomic levels appear to have a similar effect. For instance, *Bifidobacterium* in Actinobacteria, *Faecalibacterium*, *Lachnospiraceae* and *Lactobacillus* in Firmicutes, *Prevotella* and *Parabacteroides* in Bacteroidetes increased in immunotherapy‐sensitive individuals (Figure 1, Table 1).^34^, ^35^, ^36^, ^37^, ^38^ McCulloch et al. found that several Actinobacteria and *Lachnospiraceae* of Firmicutes showed a potential sensitising effect, whereas a number of *Bacteroidetes* and *Proteobacteria* showed an immunotherapy‐attenuating effect.^39^

Many strains that affect the effectiveness of immunotherapy belong to the same genus or family. The microbiota of different strains as an ensemble may be a promising marker for estimating the effects of cancer immunotherapy. However, more clinical data are required to determine which taxonomic level or individual is more appropriate. Currently, many mechanistic studies have focused on the investigation of a single strain, and it is important to explore the common mechanisms among strains with similar effects.

### 2.2 Common ‘harmful’ bacteria and extraintestinal microbes could modulate immunotherapy processes

A well‐known ‘cancer‐promoting bacterium’, *Fusobacterium nucleatum*, which was first discovered in the oral cavity, has been linked to poor prognosis in colorectal cancer (CRC).^40^, ^41^ In contrast, Gao et al. showed that *F. nucleatum* stimulated the STING signalling pathway, increases PD‐L1 expression and consequently enhanced the efficacy of anti‐PD‐L1 antibodies.^32^ Therefore, a certain ordinary ‘harmful’ bacteria may be helpful in immunotherapy.

With the development of sequencing technology, we have observed an increasing number of changes in the microbiota of ecological niches outside the gut. *Helicobacter pylori* in the stomach diminished the efficacy of immunotherapy.^25^ *Porphyromonas gingivalis*, a major pathogen involved in periodontitis, upregulated the immune checkpoint pathway and cause adverse ICB‐related effects.^42^ Recently, the microbiota present in the tumour microenvironment and within tumour cells has also come into the limelight.^43^, ^44^, ^45^, ^46^ According to Shi's work, an increase in the microbiota in tumour tissue facilitated CD47‐based immunotherapy.^44^ A recent study showed that ICIs therapy was enhanced by *Lactobacillus reuteri* in tumour tisssue.^45^ Additionally, numerous solid tumour cells have been shown to contain bacteria.^43^ A recent study showed that the bacteria in breast cancer cells can promote metastasis.^47^

### 2.3 Research requires a more rigorous experimental design and optimised methodology

Interestingly, the same strain caused inconsistent changes in the gut of patients receiving immunotherapy, as *Ruminococcus obeum*, *Klebsiella pneumoniae* and *Lactobacillus* spp. demonstrated opposing effects in various trials and malignancies (Figure 1). Additionally, a machine‐learning research has shown that the microbiota has a limited capacity for reproducibility.^46^ Additional variables that may affect the results include sampling time, sample quality, preservation conditions and sequencing techniques.^48^ For instance, McCulloch et al. discovered that the best link between microbiota composition and clinical outcomes was only seen roughly 1 year after immunotherapy began, probably because some patients had depleted their beneficial microbiota or experienced new alterations in tumour‐intrinsic factors.^39^

## 3 POTENTIAL MECHANISMS BY WHICH MICROBIOTA INFLUENCES IMMUNOTHERAPY

### 3.1 Microbial signals—Microbial direct antigenicity, metabolites and others

**FIGURE 2:**
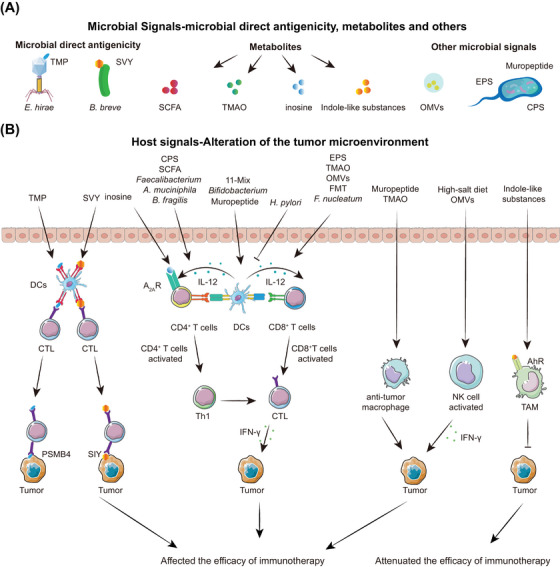
Potential mechanisms by which microbiota influenced immunotherapy. (A) Microbial signals. ① Microbial direct antigenicity, such as the antigenic epitope SVY of *B. breve* and the TMP of the *Enterococcus hirae* phage genome. ② Metabolites such as inosine, SCFA, TMAO and indole‐like substances. ③ Other microbial signals, including OMVs, EPS, CPS and muropeptide. ④ Microbes of unknown specific signals. Some microbes that could influence the efficacy of immunotherapy but whose exact composition is not yet known, such as *Faecalibacterium*, *F. nucleatum* and *H. pylori*. (B) Host signals. ① Microbial direct antigenicity was similar to that of tumour neoantigens (PMSB4 or SIY) and enhanced the killing effect of CTL by DC antigen presentation. ② Microbial signalling enhanced the efficacy of immunotherapy by promoting DCs maturation or activation, which, in turn, activated CD4^+^ T cells and CD8^+^ T cells, or by directly activating CD4^+^ T cells or CD8^+^ T cells. ③ Immune‐active muropeptides: TMAO enhanced the effect of antitumour macrophages. ④ High‐salt diet: OMVs activated NK cells to enhance their antitumour effects. ⑤ Indole‐like substances activated the AhR receptor of TAM to attenuate its antitumour effects.

The microbiota could serve as cross‐reactive tumour antigens, thus directly stimulating the host immune system.^49^, ^50^ To communicate with the host, they create signalling molecules such as metabolites,^23^, ^51^, ^52^ extracellular vesicles or polysaccharides (Figure 2A).^53^, ^54^

#### 3.1.1 Microbial direct antigenicity

The cross‐reactivity of bacterial and tumour antigens could exert antitumour effects via T helper cells or cytotoxic T lymphocytes (CTLs).^55^ Due to the similarities between the antigenic epitopes of *Bifidobacterium breve* and cancers, SVYRYYGL (SVY)‐specific T lymphocytes were able to identify a model neoantigen SIY and inhibit tumour growth.^49^ Kalaor et al. demonstrated the existence of human leukocyte antigen (HLA) peptidomic signatures originating from bacteria.^50^ In addition to bacterial antigens, phages in the gut could activate immune responses. Enterococcal bacteriophages contain the tail length tape protein (TMP), which is a binding epitope for major histocompatibility complex (MHC) class I molecules. Mice carrying this phage display a TMP‐specific CD8^+^ T cell response during anti‐PD‐1 therapy.^56^

In conclusion, new microbial antigen mining and identification are key for influencing the efficacy of immunotherapy through direct antimicrobial antigenicity. The development of multiomics and artificial intelligence provides a new opportunity to screen for new microbial antigens relevant to immunotherapy.

#### 3.1.2 Metabolites

Numerous bioactive compounds are produced by the gut microbiota as by‐products of their metabolism. High levels of faecal or plasma short‐chain fatty acids (SCFAs) are associated with improved responsiveness to immunotherapy and longer progression‐free survival (PFS).^23^, ^57^ However, two‐patient cohorts and preclinical studies were observed the opposite conclusion.^23^ In addition, the conversion of dietary tryptophan to indole by *Lactobacillus* prevented the reduction of tumour‐associated macrophage (TAM) aryl hydrocarbon receptor (AhR) activity and aggregation of intratumoural TNF‐α^+^ IFN‐γ^+^ CD8^+^ T cells, attenuating the efficacy of immunotherapy.^52^ Indole‐3‐lactic acid (ILA), which is produced by *Lactobacillus gallinarum*, could directly prevent carcinogenesis.^58^ Moreover, *Bifidobacterium pseudolongum*‐derived inosine,^59^ and trimethylamine N‐oxide (TMAO) which is a related metabolite of *Clostridium spp*,^51^ have been reported to affect immunotherapy efficacy.

#### 3.1.3 Other gut microbial features

Microbiota‐derived muropeptide, exopolysaccharide (EPS), capsule polysaccharides (CPS) and bacterial outer membrane vesicles (OMVs) are potential immunotherapy options.^14^, ^53^, ^60^, ^61^

*Enterococci* secrete orthologs of the NlpC/p60 peptidoglycan hydrolase SagA to produce an immune‐active muropeptide.^60^ The effectiveness of anti‐PD‐L1 antibodies was increased by SagA expression in non‐protective *E. faecalis* or synthetic muropeptides.^60^ EPS‐R1, a microbial EPS generated by *Lactobacillus delbrueckii* subsp. bulgaricus OLL1073R‐1, may stimulate CCR6^+^ CD8^+^ T cells in mouse Peyer's patches and improve the anticancer effects of immunotherapy.^53^ *B. fragilis*‐derived CPS provoked a homologous Th1 immune response that is dependent on interleukin‐12 (IL‐12) to increase the effectiveness of CTLA‐4 antibodies.^14^ Kim et al. found that bacterial OMVs could target and accumulate specifically in tumour tissues, inducing IFN‐γ expression of NK cells and T cells, finally establishing a long‐term memory effect on the antitumour response.^61^

### 3.2 Host signals—Alteration of the tumour microenvironment

The tumour microenvironment is intricate, with infiltration by various immune cells and mesenchymal components such as cancer‐associated fibroblasts (CAFs).^62^ Microbial signals usually activate the host immune system and trigger various host signals that affect the efficacy of immunotherapy (Figure 2B).

#### 3.2.1 Dendritic cells and CD4^+^ T cells

As the key cell population linking innate and adaptive immunity, dendritic cells (DCs) could induce T cell responses by increasing the expression of surface MHC and costimulatory molecules.^63^ Bacterial antigen‐loaded DCs may trigger T‐cell‐specific responses or interact with tumour antigens.^14^, ^27^ Signalling from *Bifidobacterium* regulated DCs activation and induced IFN‐γ^+^ CD8^+^ T cells, enhancing the efficacy of immunotherapy.^13^, ^44^ Tanoue et al. isolated a consortium of 11 bacterial strains using a similar mechanism.^64^ The use of antibiotics such as vancomycin induces systemic increases in CD8α^+^ DCs and more effective expansion of adoptive antitumour T cells.^31^ An antibiotic cocktail (ABX) reduced the number of CD11c^high^ MHC‐II^high^ DCs as well as CD86 expression and IL‐12B production in tumour‐associated DCs, diminishing the efficacy of CpG‐ODN therapy.^15^ Moreover, CPS from *Bacteroides fragilis* and *Bifidobacterium* metabolite inosine activate DCs, which then initiate a Th1 immune response, thereby enhancing immunotherapeutic efficacy.^14^, ^59^

The microbiota could also directly attract CD4^+^ T cells. In mice transplanted with non‐responder faeces, oral administration of *A. muciniphila* activated the recruitment of CCR9^+^ CXCR3^+^ CD4^+^ T cells to the tumour tissue, restoring the anti‐PD‐1 response.^27^ *Faecalibacterium* enhanced the therapeutic response to ipilimumab in patients with metastatic melanoma by increasing the proportion of CD4^+^ T cells and CD25 production in the serum and decreasing the proportion of Treg cells in the peripheral blood.^22^

#### 3.2.2 CD8^+^ T cells

The microbiota may increase the effectiveness of ICIs by stimulating CD8^+^ T cells in various ways.^65^ In addition to DC‐dependent activation of CD8^+^ T cells, microbial signals could directly increase the number of CD8^+^ T cells. For instance, *F. nucleatum* increases CD8^+^ tumour‐infiltrating lymphocytes (TILs), which improves the effectiveness of anti‐PD‐L1 therapy.^32^ In clinical studies, the combination of faecal microbial transplantation (FMT) and anti‐PD‐1 therapy activated mucosa‐associated invariant T cells (MAIT) and CD56^+^ CD8^+^ T cells in peripheral blood mononuclear cells (PBMCs), resulting in increased activation of CD8^+^ T cells at tumour sites.^66^

#### 3.2.3 Tumour‐associated macrophages and NK cells

Tumours in mice colonised with immunotherapy‐responsive patient faeces have higher populations of intratumoural neutrophils and TAMs.^26^ Muropeptides produced by *Enterococci* were immunologically active and directly trigger the antitumour responses of macrophages.^60^ The microbial metabolite TMAO promoted infiltration of TAMs, increased the proportion of TNF‐α^+^ IFN‐γ ^+^ T cells in the tumour tissues and inhibited tumour growth.^51^ In pancreatic ductal adenocarcinoma (PDAC), TMAO resulted in increased expression of co‐stimulatory markers such as MHCI, MHCII and CD86 in TAM and reformulated the tumour environment to an immune‐activated state to inhibit tumour growth.^51^, ^67^ Tryptophan‐derived microbial metabolites activate the AhR in TAMs to suppress antitumour immunity and reduce the efficacy of ICIs.^52^

High‐salt diet‐induced NK cell‐mediated tumour immunity by suppressing PD‐1 expression and increasing the level of IFN‐γ.^68^ A clinical study found that patients with non‐small cell lung cancer (NSCLC) and high microbial diversity had a higher abundance of NK cell subpopulations in the peripheral blood in response to anti‐PD‐1 therapy.^28^

#### 3.2.4 Cancer‐associated fibroblasts

In the context of cancer, CAFs are defined as fibroblasts present within or close to tumour cells.^69^ CAFs could restrict the infiltration of immune cells into malignant areas by interacting with macrophages to form a spatial structure of the tumour immune barrier (TIB).^70^ Meanwhile, CAFs could secret several key molecules such as TGF‐β, Ln‐γ2, Wnt2 and exosome molecules to resist PD‐1/PD‐L1 immunotherapy.^71^ In addition to its ability to influence the efficacy of immunotherapy, CAFs have also been found to interact with microbiota. Gut microbiota derived from a high‐fat diet could increase the levels of bile acid (BA) metabolites and activate the BAs‐Farnesoid X receptor axis, which leading to the activation of CAF‐like properties in the colon, thereby promoting tumorigenesis.^72^ In addition, *Actinomyces* in CRC were found to reside in CAFs and co‐occur with various pro‐tumour microbiota.^73^ *Bifidobacterium adolescentis* were reported to directly induce CD143^+^CAFs to suppress tumorigenesis.^74^ These studies suggest that microbiota may interact with CAFs to influence immunotherapy efficacy by altering cytokine secretion and disrupting TIB.

## 4 MECHANISMS OF BACTERIAL TRANSLOCATION UNDER IMMUNOTHERAPY

### 4.1 Changes of intratumoural bacteria

Immunotherapy may affect the composition of the intratumoural microecology and have different patterns of gut microbes translocation. *E. faecalis* had the highest abundance between the first and second dose of ICI (early phase of treatment), whereas *L. johnsonii* became dominance after the second dose of ICI.^75^ Moreover, the composition of intratumoural bacteria can be altered by supplementation of exogenous bacteria. After systemic administration of *Bifidobacterium*, *L. reuteri* and *Streptococcus*, the corresponding *Bifidobacterium, L. reuteri* and *Streptococcus* could be detected in tumour tissues by using selective plates and 16S ribosomal RNA identification.^44^, ^45^, ^76^ Although some bacterial translocation has been observed, such translocation is not realised by any bacteria and some strains have a higher propensity to translocate than others. Administration of *Escherichia coli* in mice was detected in tumours, whereas *Bifidobacterium longum* was not.^45^

### 4.2 Mechanisms of bacterial translocation

**FIGURE 3:**
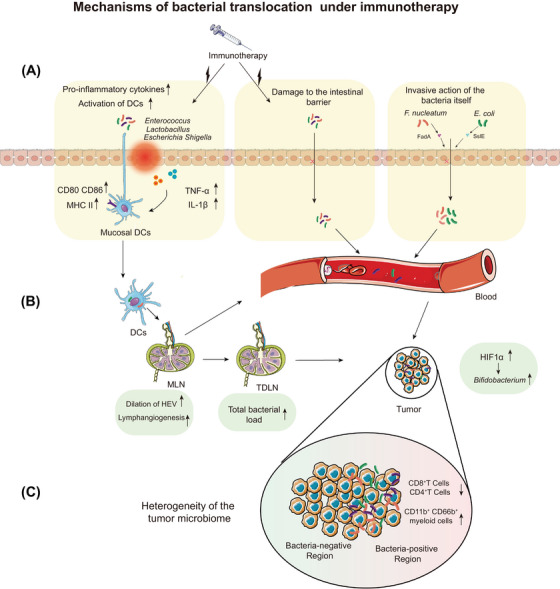
Mechanisms of bacterial translocation under immunotherapy. (A) The way that bacteria cross the intestinal barrier. Immunotherapy induced damage to the intestinal barrier or cause secretion of intestinal inflammatory factors that contribute to an increase in bacteria carried by dendritic cells. Bacteria secrete substances that break the intestinal barrier. (B) Bacteria were carried by dendritic cells into the MLN and later into the TDLN or bloodstream eventually into the tumour. And bacteria that break through the intestinal barrier can also reach the tumour after entering the bloodstream directly. (C) Intratumour bacterial distribution was heterogeneous and impacted the tumour microenvironment.

The first step in bacterial translocation is to cross the intestinal barrier (Figure 3A). *F. nucleatum* has been found to disrupt the integrity of the intestinal mucosal barrier and aggravate the inflammatory response by expressing FadA protein, which adheres to and invades host epithelial and endothelial cells.^77^ *F. nucleatum* in tumour tissues enhanced PD‐L1 expression in tumour cells and improved the efficacy of anti‐PD‐L1 immunotherapy.^32^ In addition, an *E. coli* mucinase SsLE secreted by *E. coli* promoted the translocation of its mucosal barrier.^78^ Once the intestinal barrier is compromised, the bacteria will have the opportunity to enter the bloodstream for opportunistic translocation. Interestingly, Choi et al. found that ICI promotes the translocation of gut bacteria to lymphoid organs and tumours, no ICI‐induced changes in the intestinal barrier were observed.^75^ *L. reuteri* was found to be translocated to tissues throughout the body without impair intestinal barrier integrity.^45^ Such translocation may cause by DCs which activated by ICI‐induced inflammation trafficking bacteria to the mesenteric lymph nodes (MLNs). And the process from the MLNs to tumour‐draining lymph nodes (TDLNs) and tumours is caused by enlargement of high endothelial venules and expansion of the medullary lymphatic sinus, increasing the translocation of bacteria from MLNs to the blood^75^(Figure 3B).

How could bacteria reach the extra‐intestinal site targeted (blood, lymphoid organs, other tissues and organs) to get into the tumour? Shi et al. found that *Bifidobacteria* were detected in tumour tissue but not in lung, which they hypothesised was due to the anaerobic environment in tumours being more supportive of anaerobic bacteria growth and accumulation.^44^ However, there was no significant correlation between *Streptococcus* abundance in tumour and tumour growth expression of HIF1α increased along with tumour growth.^76^ This suggests that other mechanisms may also exist, such as metabolites produced in the tumour tissue being used as navigational cues to cause localised bacterial translocation.^79^, ^80^, ^81^

It is worth noting that the distribution of bacteria that reach the tumour is not homogeneous (Figure 3C).^82^, ^83^ The composition of intratumoural bacteria varied at the phylum and genus levels even in different portions of tumour tissue from the same patient.^83^ This suggests the presence of heterogeneous spatial distribution of bacteria in the tumour tissues of some patients. Furthermore, the intratumoural bacteria were not randomly distributed, but highly organised in microniche with immune.^83^ Bacteria‐positive tissue areas had more CD11b^+^ and CD66b^+^ myeloid cells and fewer CD4^+^ and CD8^+^ T cells compared to bacteria‐negative tissue. These bacteria protect tumours from immune system attacks and help them spread throughout the body.^83^ However, the reasons for the formation of bacterial heterogeneity in tumours are still unknown.

### 4.3 Detection of bacterial translocation

Immunohistochemistry for lipoteichoic acid and lipopolysaccharide (LPS) for labelling gram‐positive and gram‐negative bacteria, selective media culture^84^ and fluorescence in situ hybridisation (FISH) techniques^85^ for a particular type of bacteria, as well as sequencing techniques and microscopic imaging have improved our ability to detect bacteria in tumours. However, the mechanism of translocation of a single particular bacterium is unclear, as there are fewer means of tracing a single bacterium. For example, most intestinal anaerobes do not have well‐developed genetic‐operating systems.^86^ With the continuous maturation of new technologies, such as fluorescently labelled D‐alanine,^87^ click chemistry labelling of the cell wall of bacteria^88^ and 5R 16S rDNA sequencing method,^43^ new means might be available for us to explore the translocation of a single bacterium.

## 5 REGULATING IMMUNOTHERAPY BY GUT MICROBIOTA MODIFICATION

The existing microbiota of patients could be modified to promote the efficacy of immunotherapy using various strategies such as antibiotic treatment, FMT, administration of specific mixed strains or single strains, prebiotics or dietary interventions. Many clinical trials are currently underway, as shown in Table 2.

**TABLE 2:** Clinical trials of altered microbiota to increase the efficacy of immunotherapy.

NCT number	Status	Cancer types	*n*	Intervention	Outcome(s) measure	Stage
Antibiotics						
NCT03785210	Completed	Hepatocellular CancerHepatocellular CarcinomaLiver MetastasisMetastatic Colorectal CancerMetastatic Pancreatic Cancer	22	Vancomycin	Best overall response (BOR), assess overall survival (OS)	Phase 2
NCT05502913	not recruiting	Metastatic lung cancer	80	Antibiotics	PFS, OS, ORR	Phase 2
NCT05462496	not recruiting	Pancreatic cancer	25	Ciprofloxacin	Achievement of overall immune response, AE, ORR, OS	Phase 2
NCT04028063	Recruiting	Advanced/metastatic soft tissue sarcoma	28	doxorubicin	PFS, OR, AE	Phase 2
NCT03817125	Completed	Metastatic melanoma	14	Vancomycin pretreatment	AEs, ORR, DCR, PFS, OS, duration of response, percentage of CD8 cells in tumour tissue	Phase 1
NCT05777603	not recruiting	Advanced NSCLC	20	aerosolized aztreonam, aerosolised aztreonam	Dose limiting toxicities (DLTs)	Phase 1
FMT						
NCT05286294	Recruiting	Solid tumours	20	FMT	Safety, FMT‐related AEs, ORR	Phase 2
NCT05279677	Not recruiting	CRC	30	FMT+Sintilimab+Fruquintinib	ORR, OS, PFS, Safety	Phase 2
NCT05273255	Recruiting	Solid tumours	30	FMT	Intestinal microbiome community change, AE, ORR, PFS, OS	NA
NCT05251389	Not recruiting	Melanoma stage III/IV	24	FMT	Efficacy (SD, PR, CR), safety, PFS, intestinal microbiome community change	Phase 1−2
NCT05008861	Not recruiting	NSCLC	20	FMT+ICI/chemotherapy	FMT‐related AEs, intestinal microbiome community change	Phase 1
NCT04951583	Recruiting	Melanoma, NSCLC	70	FMT + ICI	ORR, OS, PFS, Safety	Phase 2
NCT04924374	Recruiting	NSCLC stage III/V	20	FMT+Anti‐PD‐1	Treatment safety and responses	NA
NCT04758507	Recruiting	RCC	50	Donor/Placebo FMT + ICI	Efficacy, PFS, intestinal microbiome community change	Phase 1−2
NCT04577729	Recruiting	Melanoma stage III/IV	60	Allogenic /Autologous FMT + ICI	PFS, efficacy (SD,PR,CR)	NA
NCT04163289	Recruiting	Renal cancer	20	FMT + Nivolumab/Ipilimumab	Safety, FMT‐related AEs, ORR	Phase 1
NCT04130763	Recruiting	Gastrointestinal system cancer	10	FMT + Anti‐PD‐1	FMT‐related AEs, ORR	Phase 1
NCT03353402	Unknown	Melanoma	40	FMT + ICI	FMT‐related AEs,	Phase 1
Probiotics						
NCT05220124	Recruiting	Bladder urothelial carcinoma	190	Live combined (bifidobacterium, lactobacillus and enterococcus capsules)	PFS, duration of response (DOR), overall survival (OS), serious adverse event (SAE), overall response rate (ORR), disease control rate (DCR), intestinal microbiome community change	Phase 4
NCT04699721	Active,Not recruiting	NSCLC stage III	40	nivolumab 4.5 mg/kg+Paclitaxel (albumin‐bound type) 260 mg / m2+ Carboplatin AUC5, bifidobacterium trifidum live powder (BiFico, SINE)	AE, complications, non‐R0 surgical events, ORR, MPR, DFS, RR, OS	Phase 1
NCT05032014	Recruiting	Liver cancer	46	Lactobacillus rhamnosus Probio‐M9	ORR, PFS, OS	Not applicable
NCT05094167	Recruiting	Non‐small cell lung cancer	46	Probiotic Lactobacillus Bifidobacterium V9(Kex02)	ORR, PFS, OS	Not applicable
NCT03595683	Suspended	Melanoma	8	EDP1503	RR, PFS	Phase 2
NCT03775850	Completed	Bladder Cancer Colorectal Cancer Metastatic GastroEsophageal Cancer MSI‐H Non‐Small Cell Lung CancerRenal Cell CarcinomaTriple Negative Breast Cancer	69	EDP1503	safety, PFS, OS	Phase 1
NCT03829111	active,not recruiting	Renal cell carcinoma	30	Clostridium butyricum CBM 588 Probiotic Strain	OR, PFS, overall immune response, intestinal microbiome community change	Phase 1
NCT05576961	Recruiting	Solid tumour	14	RX‐AF01 (A. finegoldii.)	ORR, OS, PFS, DoR, change of tumour microenvironment	Phase 1,2
Prebiotics						
NCT05303493	Recruiting	Advanced non‐small cell Lung cancer Melanoma stage IV NSCLC stage IV Unresectable melanoma	41	Camu‐Camu Prebiotic	ORR	Phase 1
NCT05821751	Recruiting	Head and neck squamous cell carcinoma	40	Inulin	Intestinal microbiome community change, evaluation of immune‐phenotype, OS	Not applicable

### 5.1 Antibiotics

**FIGURE 4:**
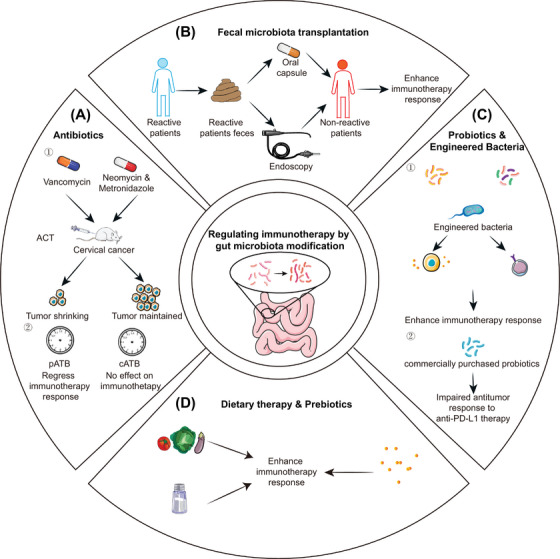
Regulating immunotherapy by gut microbiota modification. (A) Antibiotics. ① The use of vancomycin reduced tumour growth in mice with cervical cancer treated with ACT, whereas treatment with neomycin and metronidazole had no effect. ② pATB rather than cATB was associated with poorer treatment outcomes and OS. (B) FMT is a therapy that delivers faeces from a healthy donor to the recipient's gastrointestinal tract through colonoscopy or oral administration to cure the disease by restoring the balance and function of the intestinal microbiota. (C) Probiotics and engineered bacteria. Single or mixed probiotics or engineered bacteria alter the host metabolism and immunity to enhance the immunotherapy response. (D) Dietary therapy and prebiotics. High‐fibre and low‐salt diets and prebiotics influence the efficacy of immunotherapy.

Antibiotic use has also been shown to affect the efficacy of immunotherapy in clinical and preclinical studies (Figure 4A).^14^, ^15^, ^31^, ^89^, ^90^, ^91^, ^92^, ^93^, ^94^ Several studies have shown that the use of antibiotics attenuates the efficacy of ICIs in a variety of tumour types (melanoma, NSCLC, uroepithelial cancer, gastroesophageal cancer, pancreatic cell carcinoma, etc.).^89^, ^92^However, other study showed that antibiotic use improved the effectiveness of anti‐PD‐1 treatment in a mouse model of PDAC and cervical cancer receiving adoptive cell therapy (ACT).^31^, ^92^

The timing of antibiotic administration could also affect the efficacy of immunotherapy. Prospective studies have demonstrated that pATB (antibiotics prior to treatment), rather than cATB (antibiotics concurrently with treatment), are associated with poor treatment outcomes and overall survival (OS).^30^, ^89^, ^93^, ^94^ A recent study found that recolonisation with the genus *Enterocloster* with pATB resulted in the down‐regulation of MAdCAM‐1 and metastasis of α4β7^+^ TH17 and Treg17 cells from the ileum to extra‐intestinal tumours and tumour‐draining LNs, thereby reducing the efficacy of immunotherapy.^95^ Different patients may have used various types or doses of antibiotics during treatment. Owing to the complexity of antibiotic use, relatively few clinical studies have been conducted in this area. Further studies are required to determine the impact of antibiotics use throughout the immunotherapy process.

### 5.2 Faecal microbiota transplantation

FMT is a treatment that transplants microbiota from the faeces of healthy individuals into the gastrointestinal tract of patients to re‐establish a new gut microbiota that can treat intestinal and extraintestinal diseases (Figure 4B).^96^ The non‐responder phenotype to anti‐PD‐1 therapy could be altered to a responsive phenotype by FMT from responder patients in germ‐free (GF) mice model.^20^, ^21^, ^27^

Two phase I clinical trials have reported the clinical benefits of FMT in some patients with melanoma receiving immunotherapy, including CD8^+^ T cell activation and reduced numbers of IL‐8‐expressing bone marrow cells.^66^, ^97^ And adverse events caused by FMT were low‐grade in both studies.^66^, ^97^ However, many problems remain to be solved including standardising the donor screening process and determining the effective components of FMT treatment, length of the FMT procedure and methods for producing qualified donor faeces in large quantities.

### 5.3 Probiotics and engineered bacteria

Probiotics are defined as live microbes that provide health benefits to a host when consumed in adequate amounts.^98^ (Figure 4C). *Lactobacillus paracasei* sh2020 and *Bifidobacterium* alter the composition of the gut microbiota and improve the response to immunotherapy.^13^, ^59^, ^99^, ^100^ Several commercially available probiotics such as *Lactobacillus rhamnosus*, CBT and CBM588 have been explored for their role in immunotherapy.^101^, ^102^, ^103^ Nevertheless, one study discovered that PFS and patient response rates to immunotherapy with or without probiotics did not differ significantly.^104^ Meanwhile, mice receiving commercially available probiotics (*B. longum*‐ or *L. rhamnosus*‐based) exhibited an impaired antitumour response to anti‐PD‐L1 antibodies treatment.^104^ Probiotics consortia, which also influence the efficacy of immunotherapy, are symbiotic combinations of multiple bacteria. They are designed to duplicate some of the complexities of the host gut community more reliably and safely. 11‐Mix, isolated by Tanoue et al., enhanced the therapeutic effects of ICIs in a mouse tumour model.^64^ An early clinical trial applied Microbial Ecosystem Therapeutic 4 (MET4), a combination of 30 bacteria in combination with ICI in patients with advanced solid tumours. However, the results showed no significant increase in the objective and clinical response rates in the combination group.^105^ Whether the currently recognised effects of probiotics on immunotherapy are beneficial remains debatable. For these potential probiotics which are isolated the faeces of responder, researchers need to clarify the mechanisms affecting immunotherapy, and their interactions with commensal microbiota. Moreover, establishing stable long‐term coexisting probiotic consortia in which the members exert friendly interactions is a challenge.

Engineered bacteria are emerging as a hotspot in immunotherapy. NlpC/p60 peptidoglycan hydrolase SagA‐engineered probiotics have been reported to enhance anti‐PD‐L1 antitumour efficacy.^60^ Canale et al. enabled *E. coli* Nissle 1917 strains to colonise tumours and continually transform ammonia, a metabolic waste product of tumours, into L‐arginine. *E. coli* Nissle 1917 showed significant synergistic effects with anti‐PD‐L1 therapy for tumour clearance.^106^ By clarifying the bacterial composition and underlying molecular mechanisms affecting immunotherapy, the modification of engineered bacteria could contribute to the enhancement of safety and efficacy.

### 5.4 Dietary therapy and prebiotics

Drastic dietary changes could cause detectable changes in the structure of the intestinal microbiota within a relatively short period of time.^107^ Dietary patterns (e.g., plant‐based diet, Mediterranean diet) or a high fibre content affected the composition of the gut microbiota and host immune response, and significantly enhanced the efficacy of immunotherapy (Figure 4D).^46^, ^104^, ^108^, ^109^ A low‐salt diet improved the efficacy when using a suboptimal dose of anti‐PD‐1 antibodies.^68^ A choline‐rich diet inhibited tumour growth by upregulating tumour TMAO levels through the microbiota.^110^ Moreover, ginseng polysaccharides (GPs) increased the antitumour response to anti‐PD‐1 therapy by increasing the microbial metabolite valeric acid and decreasing L‐kynurenine and the Kyn/Trp ratio. Combination treatment with GPs and anti‐PD‐1 antibodies sensitised mice receiving microbiota (non‐responders became responders).^111^ The role of inulin and the polyphenol castalagin as a prebiotic in immunotherapy has also been elucidated.^104^, ^112^

## 6 INTERACTIONS BETWEEN IMMUNOTHERAPY, HOST AND GUT MICROBIOTA

### 6.1 Microbial modulation of immunotherapy‐related toxicity

**FIGURE 5:**
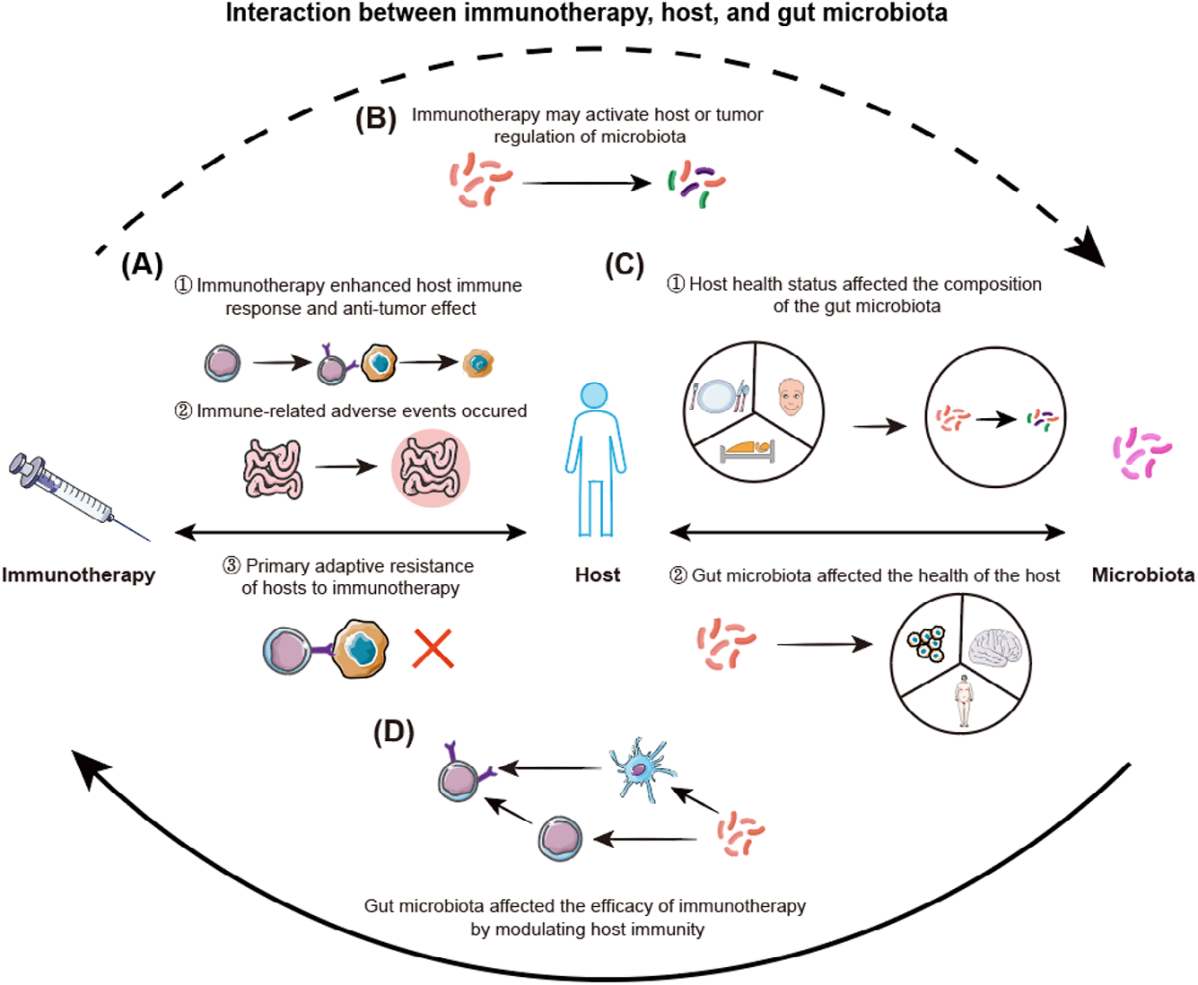
Interactions between immunotherapy, host and gut microbiota. (A) Relationship between the host and immunotherapy. ①② Immunotherapy enhances the host immune response and antitumour effect but can also cause immune‐related adverse events. ③ The host develops resistance owing to intrinsic factors. (B) Relationship between host and microbiota. ① The health status of the host, including dietary patterns, sleep, mood and primary diseases, affects the composition of the gut microbiota. ② The gut microbiota can cause various alterations in the health status of the host, including obesity, neurological changes and cancer. (C) The gut microbiota affects the efficacy of immunotherapy by modulating host immunity. (D) Immunotherapy may activate host and/or tumour regulation of the microbiota.

When ICIs trigger an immune cell attack on healthy tissues, it is known as an immune‐related adverse event (irAE) (Figure 5A). Colitis has the highest risk of patient death among ICI‐induced‐irAEs, whether treated with anti‐PD‐1, anti‐CTLA‐4, or combined immune checkpoint blockade (CICB).^113^ Two patients with immunotherapy‐associated colitis treated with FMT had reconstituted the gut microbiota, which showed complete resolution of clinical symptoms.^114^ Two microbial features rich in *Lachnospira* and *Streptococcus* were associated with favourable or unfavourable clinical responses and immune‐related adverse reactions, respectively.^39^, ^115^ The use of probiotics also reduces the likelihood of developing colitis caused by immunotherapy. For example, *Bifidobacterium* and *L. reuteri* attenuated ICI‐induced colitis.^116^, ^117^ 11‐Mix improved ICI therapy while eliminating colitis‐causing side effects.^64^

Many microbiota predictors of ICI monotherapy appear to predict the response, resistance and toxicity of CICB.^24^ Uncertainty persists regarding whether the microbiota that improved treatment response were the same as those of individuals who were predisposed to ICI‐associated colitis. And most existing investigations are clinical rather than preclinical studies because of the lack of a consistent preclinical study model.

### 6.2 Effect of immunotherapy on the characteristics of the gut microbiota

Numerous previously described strategies affect the gut microbiota; hence, it would be interesting to determine whether immunotherapy itself alters the microbiota (Figure 5B).

Anti‐PD‐1 therapy showed a weak but significant correlation with the composition of the microbial community.^101^ During the anti‐PD‐1 therapy, *Proteobacteria* increased from week 3 and became predominant at week 12 in non‐responders. This alteration resulted from *E. coli* enrichment. Although the gut microbiota of responders remained relatively stable at the phylum level, *Lactobacillus*, *Rumenococcaceae* and *Akkermansia muciniphila* increased.^33^ Moreover, some patients with metastatic melanoma with the gut microbiota defined as cluster B, which was not significantly responsive to anti‐CTLA‐4, were converted to cluster C, which was considerably responsive, after receiving anti‐CTLA‐4 antibodies treatment. Cluster C showed growth of *B. fragilis*, which increased the effectiveness of anti‐CTLA‐4 antibodies, indicating that anti‐CTLA‐4 therapy could change the prevalence of immunogenic *Bacteroides*.^14^ Immunotherapy may alter the composition of the gut microbiota; however, how this occurs remains unknown. This makes it possible to manipulate the gut microbiota in a manner beneficial for immunotherapy.

### 6.3 Interrelationship between host, gut microbiota and immunotherapy

Host, gut microbiota and immunotherapy are interconnected. The host's overall health, including dietary habits, sleep patterns, mood and major diseases, leads to changes in the metabolism and immune system that alter the gut microbiota composition.^118^, ^119^ The gut microbiota could cause various alterations in the health status of the host, including obesity, neurological changes,^118^, ^119^, ^120^ promotion or inhibition of cancer development and antitumour factors (Figure 5C).^121^

Immunotherapy promotes antitumour effects when the host is suffering from cancer by enhancing the immune response.^122^ The host itself could also develop immunotherapy resistance owing to factors intrinsic to primary or adaptive resistance, including a lack of antigenic mutations, deletion of tumour antigen expression, deletion of HLA expression and altered antigen processing mechanisms.^123^ Because key negative regulators of T cells function were removed, the use of immunotherapy could also lead to irAEs (Figure 5A).^124^

Through the aforementioned microbial signals, the gut microbiota affects the host's immune system and how immunotherapy works. Additionally, the host's resistance to immunotherapeutic toxicity and sensitivity are affected by the microbiota (Figure 5D). Conversely, immunotherapy activated the host regulation of the microbiota, enhancing or attenuating the antitumour efficacy of immunotherapy by altering the composition of the gut microbiota (Figure 5B).

In both healthy and diseased states, the host and gut microbiota influence each other. The gut microbiota, as an environmental factor, influences the host's responsiveness to immunotherapy by affecting the host's immune status, in conjunction with the host's own genetic factors. The side effects of immunotherapy are regulated by the gut microbiota and are host‐related. Furthermore, changes in the gut microbiota driven by immunotherapy have been noted in several trials; however, further investigation is required.

## 7 CONCLUSIONS

The influence of microbiota on immunotherapy is complex and varied, with different species having different effects on immunotherapy. The microbiota influences the efficacy of immunotherapy by producing metabolites, antigen mimicry and other microbial signals that induce changes in the host tumour microenvironment. The gut microbiota could translocate to tumour tissue under conditions of immunotherapy. The extraintestinal microbiota also has an impact on the efficacy of immunotherapy. Antibiotics, FMT and other means of regulating the microbiota offer new strategies for combination therapy with immunotherapy.

In recent years, many reviews have described the relationship between gut microbiota and immunotherapy from various perspectives.^125^, ^126^ Although the complex relationship between gut microbiota, host and immunotherapy has been consistently revealed, the specific modes of co‐operation and mechanisms of action between the three need to be further elucidated. Moreover, current microbiota research focuses on the intestinal tract, with infinite possibilities for the microbiota in extraintestinal organs and tumours. The components of the tumour microenvironment are intricate. The role of the gut microbiota on mesenchymal components such as tumour‐associated fibroblasts, in addition to mediating the host immune system, is in its preliminary stages.

Here, we present an illustration of the interactions between the microbiota, host and immunotherapy. The presence of microbiota in the host causes microbial signals that affect responsiveness to immunotherapy and side effects. The biological status and behaviour of the host  equally induce changes in the microbiota. In the context of immunotherapy, alterations in the gut microbiota have been observed along with the changes in the host immune system; however, it is still unknown how these changes affect the host. In addition, we emphasise the role of extraintestinal bacteria in immunotherapy. The presence and role of bacteria in tumour cells makes their impact on immunotherapy an interesting direction as well. Moreover, CAFs may interact with immunotherapy efficacy under the influence of bacteria in the tumour microenvironment. And we note changes in the intratumoural bacteria in the context of immunotherapy and summarise the possible mechanisms by which the bacteria translocate to the tumour. Although the utilisation of the microbiota as a strategy for cancer monotherapy or as an accessory to first‐line therapy has undergone extensive investigation, there remain several aspects that deserve further refinement and exploration with regard to administration and clinical translation.

As an increasing number of studies are being conducted, the development of gut microbiota as a therapeutic tool may provide additional opportunities to improve immunotherapy efficacy, and it is foreseeable that the microbiota will become an integral part of cancer treatment, given the growing evidence that the microbiota affects treatment outcomes.

## AUTHOR CONTRIBUTIONS

Yao Jiang, Dingjiacheng Jia and Yong Sun collected the data. Yao Jiang and Dingjiacheng Jia drafted the manuscript. Ning Ding and Liangjing Wang supervised and revised the manuscript. These authors contributed equally: Yao Jiang and Dingjiacheng Jia.

## CONFLICT OF INTEREST STATEMENT

All authors declare no potential conflicts of interest.

## ETHICAL STATEMENT

Not applicable.

## Data Availability

Not applicable
